# Polar Organizing Protein PopZ Is Required for Chromosome Segregation in Agrobacterium tumefaciens

**DOI:** 10.1128/JB.00111-17

**Published:** 2017-08-08

**Authors:** Haley M. Ehrle, Jacob T. Guidry, Rebecca Iacovetto, Anne K. Salisbury, D. J. Sandidge, Grant R. Bowman

**Affiliations:** Department of Molecular Biology, University of Wyoming, Laramie, Wyoming, USA; Philipps-Universität Marburg

**Keywords:** Agrobacterium, PopZ, cell division, cell polarity, chromosome segregation

## Abstract

Despite being perceived as relatively simple organisms, many bacteria exhibit an impressive degree of subcellular organization. In Caulobacter crescentus, the evolutionarily conserved polar organizing protein PopZ facilitates cytoplasmic organization by recruiting chromosome centromeres and regulatory proteins to the cell poles. Here, we characterize the localization and function of PopZ in Agrobacterium tumefaciens, a genetically related species with distinct anatomy. In this species, we find that PopZ molecules are relocated from the old pole to the new pole in the minutes following cell division. PopZ is not required for the localization of the histidine kinases DivJ and PdhS1, which become localized to the old pole after PopZ relocation is complete. The histidine kinase PdhS2 is temporally and spatially related to PopZ in that it localizes to transitional poles just before they begin to shed PopZ and disappears from the old pole after PopZ relocalization. At the new pole, PopZ is required for tethering the centromere of at least one of multiple replicons (chromosome I), and the loss of *popZ* results in a severe chromosome segregation defect, aberrant cell division, and cell mortality. After cell division, the daughter that inherits polar PopZ is shorter in length and delayed in chromosome I segregation compared to its sibling. In this cell type, PopZ completes polar relocation well before the onset of chromosome segregation. While A. tumefaciens PopZ resembles its C. crescentus homolog in chromosome tethering activity, other aspects of its localization and function indicate distinct properties related to differences in cell organization.

**IMPORTANCE** Members of the Alphaproteobacteria exhibit a wide range of phenotypic diversity despite sharing many conserved genes. In recent years, the extent to which this diversity is reflected at the level of subcellular organization has become increasingly apparent. However, which factors control such organization and how they have changed to suit different body plans are poorly understood. This study focuses on PopZ, which is essential for many aspects of polar organization in Caulobacter crescentus, but its role in other species is unclear. We explore the similarities and differences in PopZ functions between Agrobacterium tumefaciens and Caulobacter crescentus and conclude that PopZ lies at a point of diversification in the mechanisms that control cytoplasmic organization and cell cycle regulation in Alphaproteobacteria.

## INTRODUCTION

Many rod-shaped bacteria are polarized, meaning that one end of the cell is different from the other. In species that exhibit unipolar flagella or stalks, polarization is plainly apparent at the morphological level (1). Importantly, bacterial cell polarization is also apparent in the distribution of molecules in the cytoplasm. When the asymmetrically localized factors have regulatory functions, such as the control of transcription or the timing of chromosome replication, cell division produces daughter cells with distinct physiologies. For example, asymmetric cell division in the alphaproteobacterium Caulobacter crescentus produces a smaller, flagellated cell that is delayed in chromosome replication and has a different pattern of gene expression than its sibling, which is longer and replicates its chromosome immediately after cell division (2).

Decades of intensive research on C. crescentus has revealed the poles to be highly complex regions that include many different regulatory proteins, including histidine kinases, response regulators, transcription factors, proteases, protease adaptors, and others that provide a wide range of influences on cellular control (3). Many of these proteins are conserved in Alphaproteobacteria (4), and there is good evidence that at least some aspects of their function are also conserved. Brucella abortus (5), Agrobacterium tumefaciens (6, 7), Sinorhizobium meliloti (8), and Ruegeria (9) express homologs that are asymmetrically localized to the cell poles, and the phenotypes of gene knockouts suggest that they play roles in cell cycle regulation and cell polarity. However, in nearly all of these cases, specific knowledge of protein function is limited to the C. crescentus model, and the relatively low number of known polar proteins in other alphaproteobacteria makes it difficult to gain an understanding of the connectivity of polar networks in these species.

One conserved category of regulatory proteins is histidine kinases. C. crescentus produces at least four histidine kinases that participate in a complex mechanism for regulating the timing of chromosome segregation (10), and each of these is localized to one or both poles at some stage in the cell cycle. Another group of conserved factors acts directly in chromosome segregation and polar anchoring of the chromosome centromere. At the core of the segregation mechanism is a small set of repeated DNA sequences called *parS* sites, which are recognized by the protein ParB. Oligomerization of ParB forms a localized cluster of protein and DNA known as the centromere (11), which serves as the lead segment of DNA during chromosome segregation (12). In C. crescentus, the unidirectional movement of the centromere across the cell ends with its tethering to the cell pole. This is accomplished by a direct interaction between ParB and other components of the centromere segregation machinery with a pole-localized protein called PopZ (13, 14, 15).

When rod-shaped bacteria divide, new cell poles are formed at the site of the division plane. C. crescentus PopZ accumulates at the new cell pole during chromosome segregation. Thus, the delivery of the centromere is coincident with the placement of the polar tether PopZ (16). In C. crescentus, PopZ is also localized to the opposite, or “old,” pole, and here it does not appear to form a stable tether with the centromere (17). Instead, PopZ at the old pole is important for the localization of at least seven polar regulatory proteins (17, 18). The list includes two of the polar histidine kinases that are important for cell cycle control. Because of its multifaceted role in bringing proteins to C. crescentus cell poles, PopZ is called a polar organizing protein.

In this work, we assess the function of the PopZ homolog in Agrobacterium tumefaciens. Recent studies using a green fluorescent protein (GFP)-tagged fusion protein have shown that A. tumefaciens PopZ is localized exclusively to the new pole and disappears from the old pole shortly after cell division (7, 19). A similar pattern was observed for the PopZ homolog in Brucella abortus (20). This localization pattern is quite different from what is observed in C. crescentus and strongly suggests that PopZ does not have the same set of polar organizing functions in all alphaproteobacterial species. The taxonomic class Alphaproteobacteria is known for having unusually large diversity in genome size, environmental distribution, and metabolic strategies, and because the species have evolved adaptations for so many different environments, they have been called the “Darwin finches” of the bacterial world (21). The large multiprotein complexes at cell poles are an intersection point for factors that control the cell cycle and the production of morphological features, such as stalks and flagella, and therefore may be key anatomical features in species adaptation.

To determine the function of A. tumefaciens PopZ, we created a *popZ* knockout strain and characterized the mutant phenotype. Using the C. crescentus model as a basis for comparison, we asked if the knockout strain was defective in tethering the centromere of chromosome I and in the polar localization of three histidine kinases. We also complemented the *popZ* deletion by expressing a fluorescent mChy-PopZ fusion protein from the native *popZ* promoter, and this allowed us to ask if PopZ colocalizes with the centromere of chromosome I or the three histidine kinases. Further, we asked if polar asymmetry in the distribution of PopZ and the histidine kinases is correlated with differences in the timing of chromosome I replication and segregation. Overall, we find several points of similarity in the functions of C. crescentus and A. tumefaciens PopZ, including a role for PopZ in the anchoring of chromosome centromeres to the cell pole. However, we also found that these species have significant differences in the dynamic localization of PopZ and related aspects of polar organization, suggesting that the mechanisms responsible for cellular organization have undergone adaptation to suit species-specific cell anatomies.

## RESULTS

### Loss of *popZ* results in abnormal cell division and ectopic budding.

**FIG 1:**
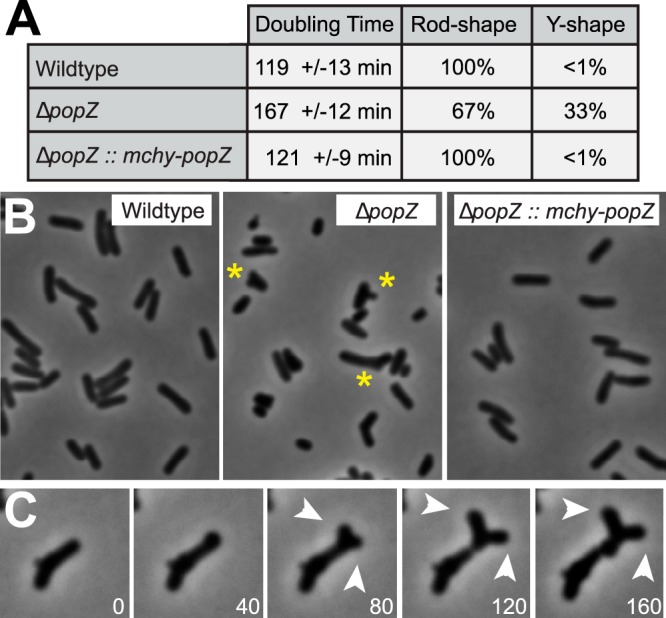
Growth characteristics of wild-type and Δ*popZ* cells. (A) The table records average doubling times of the indicated strains growing at exponential phase in ATGN medium at 30°C. Through observation by phase-contrast microscopy, cells with sidewall extensions or forked poles were distinguishable from normal rod-shaped cells and counted as Y-forms. Data were collected from >250 cells, counting 40 to 60 individuals from two representative fields in three separate experiments. (B) Phase-contrast images of cells growing at exponential phase. Examples of Y-form cells are marked by asterisks. (C) A time course showing a Δ*popZ* cell elongating and splitting at the growth pole to produce two growth poles (arrowheads). Phase-contrast images are shown in grayscale and time is displayed in minutes.

To determine the role of PopZ in Agrobacterium tumefaciens, we created a Δ*popZ* knockout strain in which the coding sequence of *popZ* (locus ATU1720) was replaced with a genetic cassette bearing spectinomycin antibiotic resistance. We found that the doubling time of the Δ*popZ* strain was 40% longer than that of the parent strain in liquid cultures (Fig. 1A). Whereas wild-type A. tumefaciens cells elongate by the extension of cell wall at budding sites that form at the new pole after cell division (22), Δ*popZ* cells displayed a range of aberrant morphologies that suggest defects in budding and/or cell division, including branched Y-forms, lumpy side walls, and aberrant cell lengths (Fig. 1A and B). Time-lapse movies of Δ*popZ* cells revealed that shortened cells are often formed by cell division events that take place close to the growing pole, and that Y-forms occur by the splitting of a growth pole into two separate growing poles (Fig. 1C; also see Movie S1 in the supplemental material). A more detailed analysis of the effects of this mutation on cell wall formation and cell morphology is published in a companion study (23). Overall, we conclude that A. tumefaciens popZ is required for normal cell growth, and that the loss of *popZ* results in abnormal cell division and ectopic bud formation.

### PopZ is dynamically relocalized from old to new pole during cell division.

**FIG 2:**
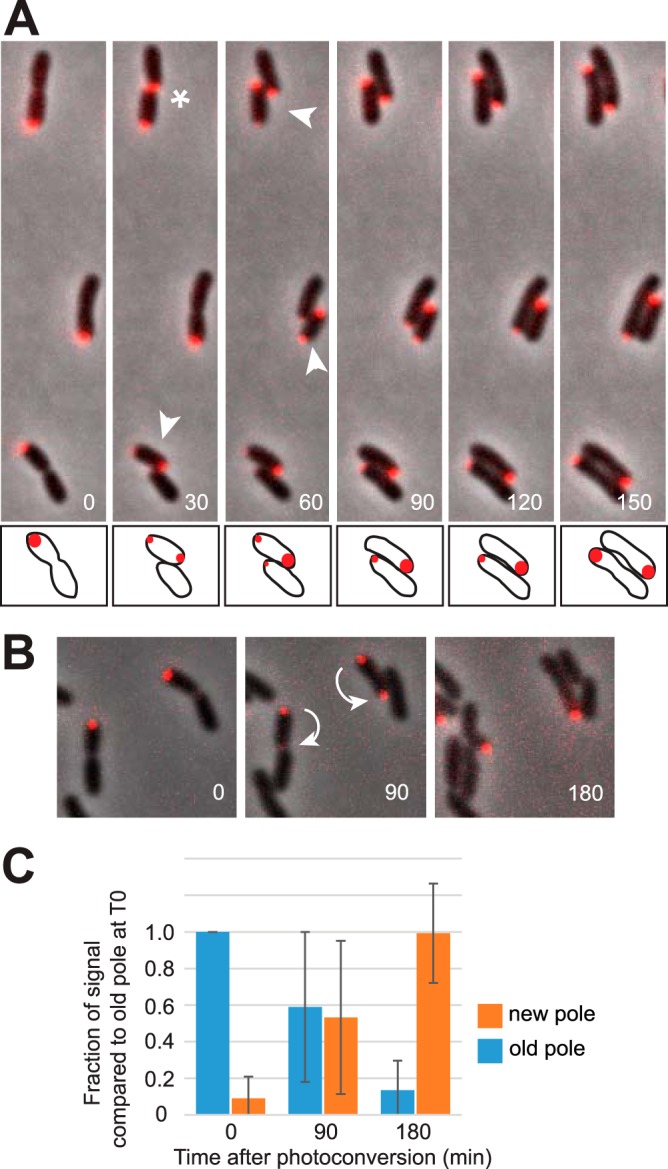
Dynamic subcellular localization and long-term stability of PopZ. (A) Time-lapse images of cells expressing mChy-PopZ (red), overlaid on a phase-contrast background (grayscale), with time displayed in minutes. Arrowheads indicate newborn daughter cells in which mChy-PopZ foci are relocated from old pole to new pole. The asterisk indicates a cell in which mChy-PopZ accumulates at the site of cell division before the daughter cells are clearly separated. For clarity, the lower cell is drawn in cartoon form at the bottom of the images. (B) Time-lapse images showing the fate of mEos3.2-PopZ after photoconversion into the red form at the 0-min time point (T0). Arrows indicate the transition from old pole to new pole. (C) Quantitative analysis of mEos3.2-PopZ localization dynamics. We identified 20 cells that divided and completed the transition in mEos3.2-PopZ localization during a 180-min time course in each of three separate experiments. For each cell, the fluorescence intensity of photoconverted mEos3.2-PopZ at the new pole or the old pole at the indicated time was divided by the intensity of photoconverted mEos3.2-PopZ at the original pole at T0. The chart shows average values and standard deviations from the means.

To determine when and where PopZ is present during the cell cycle, we placed an *mChy-popZ* coding sequence downstream of the chromosomal *popZ* promoter in the Δ*popZ* strain, making this the only copy of *popZ* in the cell. This allowed us to observe the subcellular localization of mChy-PopZ when expressed from its native promoter. This strain did not have the morphological defects found in Δ*popZ* cells, and both cell length and growth rate were indistinguishable from those of the wild type, suggesting that the fluorescent protein fusion is fully functional (Fig. 1A and B). As reported for plasmid-based expression of PopZ-GFP (7), we found that mChy-PopZ is localized to a single cell pole, and that during cell division it undergoes a dramatic redistribution from the old pole to the new pole formed by the division plane (Fig. 2A, arrowheads; Movie S2). The daughter cell that does not inherit a bright focus of mChy-PopZ from its mother slowly accumulates mChy-PopZ at the new cell pole as the cell elongates (Fig. 2A, arrows). In a minority of cells, mChy-PopZ appears in the area of the new poles before the daughter cells have clearly separated (Fig. 2A, asterisk), making it difficult to distinguish polar targeting from accumulation at the division plane.

The localization pattern of mChy-PopZ in A. tumefaciens is particularly striking compared to that of C. crescentus, in which a bright focus of PopZ is stably maintained at the old pole following cell division (17). One mechanism by which A. tumefaciens PopZ could be eliminated from the old pole is by localized proteolysis (24). Alternatively, it could be relocated to the opposite pole during cell division. To distinguish between these possibilities, we followed a cohort of PopZ molecules by expressing them as a fusion with the photoconvertible fluorescent protein mEos3.2 (Fig. 2B). A 10-s exposure to blue light at the beginning of the time series converted mEos3.2-PopZ from green to red fluorescence. On average, 99% of the photoconverted mEos3.2 was transferred to the new poles after cell division (Fig. 2C), indicating that PopZ is not eliminated through proteolysis, and nearly all of it is relocated to the opposite cell pole after cell division. Notably, we never observed the transfer of photoconverted mEos3.2-PopZ into the daughter cell opposite the mEos3.2 polar focus. This suggests that the PopZ that accumulates in the area of the division plane in cells that have not clearly separated is wholly partitioned into the daughter cells that initiated cell division with a bright polar focus of mEos3.2-PopZ at the old pole. Further, the polar foci of PopZ that slowly accumulate in the other daughter cell must be derived from *de novo* synthesis.

### PopZ relocalization is accompanied by turnover in polar histidine kinases.

**FIG 3:**
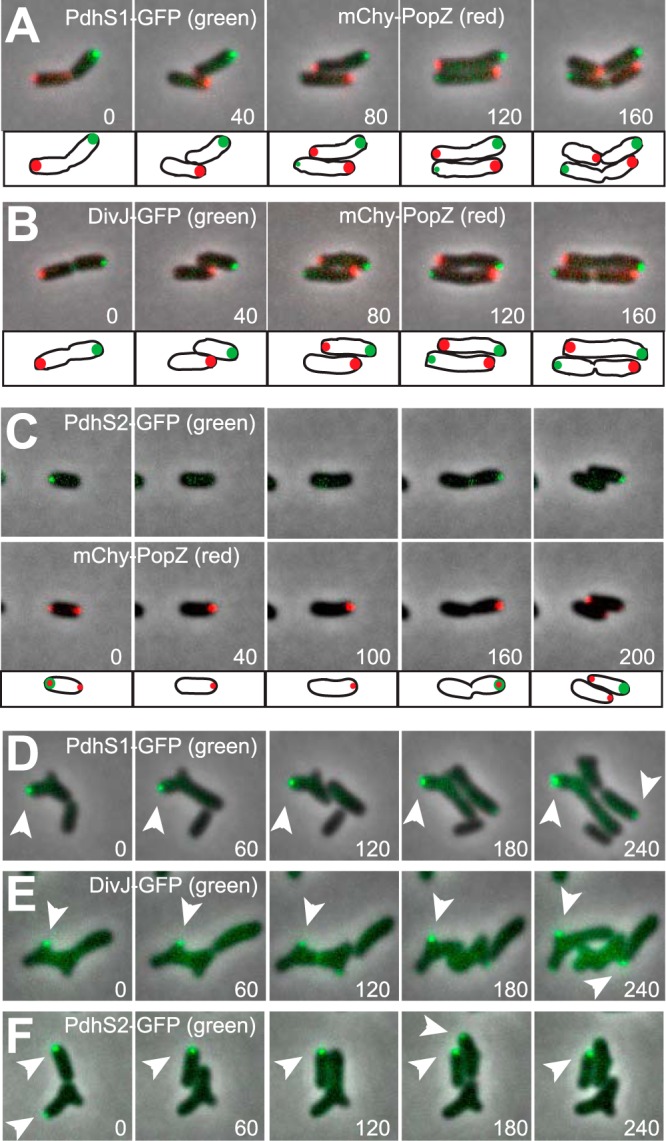
Subcellular localization of polar regulator proteins in wild-type and Δ*popZ* mutants. (A to C) PdhS1-GFP, DivJ-GFP, or PdhS2-GFP was coexpressed with mChy-PopZ in wild-type cells, and the localization patterns were observed by time-lapse fluorescence microscopy. In panel C, red and green fluorescence channels from the same dividing cells are compared in separate image sequences. For clarity, the cells are drawn in cartoon form at the bottom of the image panels. (D to F) PdhS1-GFP, DivJ-GFP, or PdhS2-GFP was expressed in Δ*popZ* cells, and localization was observed by time-lapse fluorescence microscopy. Arrowheads indicate polar fluorescent foci. In all panels, each image shows an individual frame from a time-lapse series, with time in minutes shown. Fluorescence images (in red and green) are overlaid on a phase-contrast background.

To better understand the cell cycle mechanisms that drive PopZ dynamics, we screened several candidate proteins for subcellular localizations that correlated in time and space with mChy-PopZ. One of our approaches was to determine the localization of A. tumefaciens histidine kinases that are most similar in amino acid sequence to C. crescentus histidine kinases that require *popZ* for polar localization (17). We labeled three of these kinases, PdhS1 (ATU0614), DivJ (ATU0921), and PdhS2 (ATU1888), by expressing them from a low-copy-number plasmid as fusion proteins with monomeric superfolder GFP at the C terminus. Although we do not know if the fusion proteins are fully functional, we did observe clear polar localization patterns. Time-lapse microscopy showed that PdhS1-GFP and DivJ-GFP accumulate at old poles after PopZ has been redistributed to the new pole, and that they are stably maintained at the old pole through subsequent cycles of cell growth and division (Fig. 3A and B; Movie S3).

PdhS2-GFP exhibited dynamic localization (Fig. 3C; Movie S4). It colocalized with mChy-PopZ at the old pole, during the transitional period in which mChy-PopZ was undergoing polar relocation. PdhS2-GFP remained localized at the old pole through the process of mChy-PopZ relocation and exhibited diffuse localization after relocalization was complete. After a period of cell elongation, PdhS2-GFP polar foci reappeared in late predivisional cells, colocalizing with mChy-PopZ just before the next round of cell division and the onset of mChy-PopZ relocalization. The results indicate that PopZ relocalization is correlated with a temporary change in the localization of polar signaling proteins, although the precise role of PdhS2 is unclear. Unlike Δ*popZ* strains, Δ*pdhS2* strains are wild type in morphology and growth rate (6), suggesting that PdhS2 is not upstream of PopZ activity.

### DivJ, PdhS1, and PdhS2 do not require PopZ for polar localization.

We asked if the localization patterns of the three histidine kinases were altered in the Δ*popZ* strain. PdhS1-GFP and DivJ-GFP both exhibited polar localization in the Δ*popZ* background, and they formed new foci even in branching cells with unusual cell divisions (Fig. 3D and E; Movie S5). Notably, PdhS1-GFP and DivJ-GFP foci tended to be stable over multiple cell divisions, and they did not occur at new poles or bud sites that were actively growing. We conclude that DivJ-GFP and PdhS1-GFP do not require initiation by PopZ for targeting to old cell poles. We found that PdhS2-GFP retains a pattern of transient polar localization in Δ*popZ* cells (Fig. 3E; Movie S6), often appearing at a pole before cell division and disappearing minutes later. Thus, the localization of this polar marker is also independent of PopZ.

### Agrobacterium tumefaciens PopZ is required for chromosome segregation.

**FIG 4:**
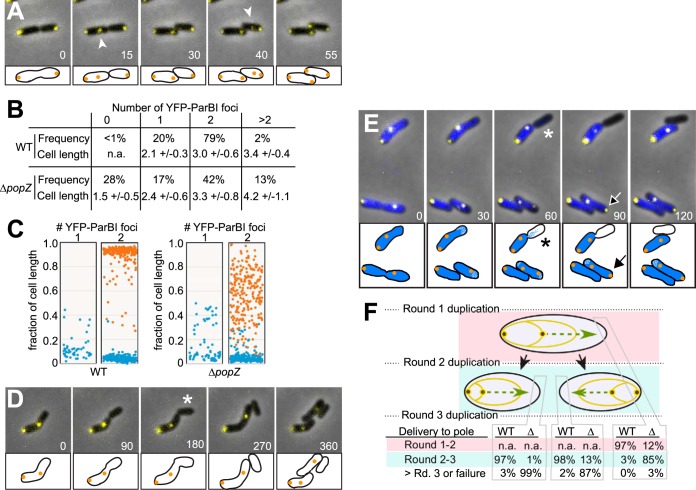
Dynamic localization of chromosome I centromeres in wild-type and Δ*popZ* mutant cells. (A) YFP-ParBI expression was used to track the position of chromosome I centromeres in wild-type cells in a time-lapse image series. Arrowheads point to YFP-ParBI foci that are moving toward the new cell poles, indicating chromosome I segregation. (B) The frequency distribution of the number of YFP-ParBI foci in wild-type and Δ*popZ* cells. Corresponding cell lengths with standard deviations are indicated. (C) The locations of YFP-ParBI centromeres were plotted as a function of cell length in cells with one and two foci. Blue points indicate the positions of centromeres nearest to a cell pole, and orange points indicate the position of the other centromere. (D and E) Time-lapse image series showing YFP-ParBI localization in Δ*popZ* cells. For panel E, time-lapse microscopy was performed on cells in which total DNA was labeled with DAPI (blue). Asterisks indicate daughter cells that fail to inherit a YFP-ParB focus and, in panel E, very little total DNA. The arrow points to an old pole that acquires a YFP-ParBI focus after centromere duplication. (F) Quantitative analysis of YFP-ParBI localization in time-lapse experiments. In panels A, D, and E, individual frames from time lapse series are shown, with time in minutes displayed. Fluorescence images are overlaid on a phase-contrast background. For clarity, the cells are drawn in cartoon form at the bottom of the images. Quantitative data shown in panels B, C, and F were collected from >250 cells, counting 40 to 60 individuals from two representative fields in three separate experiments.

Caulobacter crescentus PopZ is required for the anchoring of chromosomal centromeres to cell poles during chromosome segregation (13, 14). To observe centromere localization in Agrobacterium tumefaciens, we expressed an N-terminal yellow fluorescent protein (YFP)-tagged variant of the protein coding sequence at locus ATU2828 (located on chromosome I), which has 71% amino acid sequence similarity to the C. crescentus centromere binding protein ParB. The ParB homologs encoded by the other A. tumefaciens replicons have substantially lower levels of similarity to C. crescentus ParB. We expressed A. tumefaciens YFP-ParBI under the control of an inducible promoter on a low-copy-number plasmid (25). In wild-type cells, YFP-ParBI was visible as one or two distinct YFP foci, which were usually localized to the cell poles (Fig. 4A). At cell division, each daughter cell inherited one polar YFP-ParBI focus, and minutes later the YFP-ParBI foci were duplicated. One of the two foci moved across the cell to the new pole while the other remained in place at the old pole. This dynamic localization pattern has been observed for ParB-labeled chromosomal centromeres in C. crescentus (26) and appears to be a typical mode of chromosome segregation in proteobacteria (20, 27). Furthermore, the ATU2828 locus is in close proximity (8.5 kb) to the chromosomal origin of replication, like *parB* genes in many species (28), and fluorescence hybridization experiments have shown that the chromosome I *ori* sequence usually is located near the cell poles in A. tumefaciens (29). We therefore conclude that locus ATU2828 is the *parB* gene for A. tumefaciens chromosome I, which is segregated by a typical ParABS segregation system.

We observed aberrant patterns of ParBI centromere partitioning in A. tumefaciens Δ*popZ* strains (Fig. 4B). Compared to wild-type cells, which had either 1 or 2 YFP-ParBI foci, mutant populations included significant fractions of cells with zero or greater than two foci. The localization of YFP-ParBI foci was also defective in the Δ*popZ* mutant. Whereas the great majority of wild-type cells with two YFP-ParBI foci exhibited pole-localized centromeres, Δ*popZ* mutants exhibited one polar YFP-ParBI focus and one undocked focus at a random location in the cytoplasm (Fig. 4C). Time-lapse images of Δ*popZ* cells (Fig. 4D; Movie S7) showed that ParBI centromeres were duplicated after cell division, but the translocating copy often failed to complete translocation to the new pole, a phenotype also observed in C. crescentus Δ*popZ* strains (13, 14). Notably, A. tumefaciens Δ*popZ* cells often divided between the open pole and the missegregated centromere, creating cells that lacked a ParBI centromere. 4′,6-Diamidino-2-phenylindole (DAPI) staining revealed that these aberrant daughter cells often inherited very little total DNA, indicating a severe defect in chromosome inheritance (Fig. 4E, top; Movie S7). This explains why 28% of cells of Δ*popZ* cultures have no ParBI centromeres (Fig. 4B) and why these cells are unable to grow in time-lapse experiments (Movie S7). Zero percent of DAPI-stained wild-type cells exhibited a bulk DNA segregation defect or a failure to inherit a YFP-ParBI centromere.

To understand the dynamic behavior of YFP-ParBI centromeres over multiple generations, we tracked their localization patterns in time-lapse experiments (Fig. 4F). In wild-type cells, the translocating ParBI focus attained polar localization before the next round of cell division and chromosome duplication at 97% frequency, but this only occurred at 12% frequency in Δ*popZ* cells. Surprisingly, nearly all of the undocked centromeres in Δ*popZ* cells attained polar localization soon after chromosome duplication in daughter cell progeny (Fig. 4F and E, bottom). As a result, these cells recapitulate the most commonly occurring cell type in the population: one polar ParBI centromere and one undocked centromere (Fig. 4B and C). Notably, the recovery of polar localization only occurred after the destination pole had transitioned from a new pole (defined as the pole formed from the most recent round of cell division) to an old pole. As old poles do not accumulate PopZ, even in wild-type A. tumefaciens (Fig. 2), the simplest explanation is that old poles include a ParBI docking mechanism that does not depend on PopZ. Interestingly, PopZ accumulates at new and old poles in C. crescentus, and in Δ*popZ* mutants of this species, centromeres are undocked from both locations. Thus, A. tumefaciens may possess a centromere docking mechanism at the old pole that does not exist in C. crescentus.

### Asymmetric inheritance of mChy-PopZ is correlated with differences in the timing of chromosome segregation.

**FIG 5:**
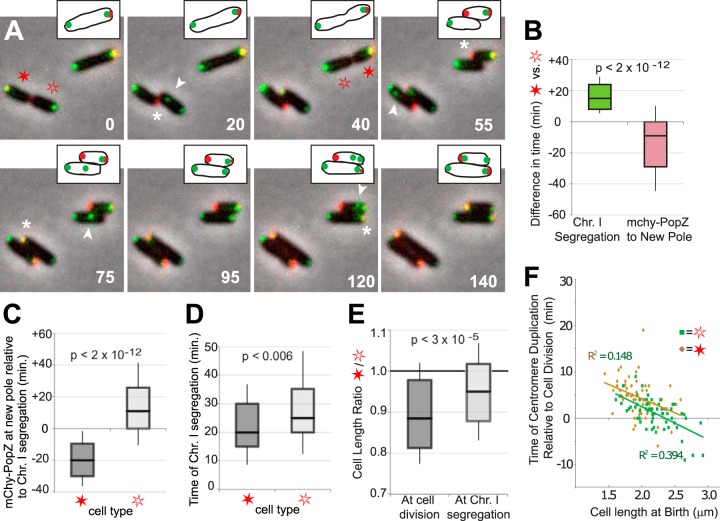
Analysis of chromosome I segregation with respect to dynamic PopZ localization and cell length. (A) Cells expressing YFP-ParBI (green) and mChy-PopZ (red) were observed by time-lapse fluorescence microscopy. Fluorescence images were overlaid on a phase-contrast image (grayscale), and time in minutes is displayed. For clarity, the upper cell is drawn in cartoon form at the tops of the images. Cells that inherit a bright focus of mChy-PopZ (closed red stars) are distinguished from their siblings (open red stars). For each cell, the segregating centromere (arrowhead) and the first appearance of a clear mChy-PopZ focus at the new cell pole (asterisk) are indicated. (B) Box plots showing the difference in the time of initiation of chromosome I segregation (green) and in the appearance of a distinct focus of mChy-PopZ at the new pole (red) between the two distinct daughter cell types. Initiation of chromosome segregation was marked as the first time frame that showed two YFP-ParBI foci. For mChy-PopZ, a distinct focus was scored if at least four adjacent pixels had intensity values that were higher than local background noise. (C) Box plots showing the difference in time between the appearance of distinct mChy-PopZ foci at the new pole and the initiation of chromosome I segregation in the two distinct daughter cell types. A negative value indicates that chromosome segregation was observed after the appearance of a polar focus of mChy-PopZ. (D) Box plots showing the length of time between the initiation of chromosome centromere I segregation and the arrival of one copy of centromere I at the new pole. (E) Box plots showing the cell length ratio for the two daughter cell types at the time of the initiation of chromosome I segregation and at cell division. (F) A scatter plot showing the relationship between cell length at birth and the time until the initiation of chromosome I segregation for the two distinct daughter cell types. Linear regressions and their associated *R*-squared values are indicated. For the box plots shown in panels B to E, 20 cells were measured from each of three separate time-lapse experiments. The midlines in the boxes indicate median values, the top and bottom edges of the boxes encompass the first and third quartiles of the data points, and whiskers mark one standard deviation from the sample mean. *P* values from a 1-tailed paired-value Student *t* test indicates that the differences are statistically significant.

We asked if the asymmetric distribution of mChy-PopZ is correlated with differences in the timing of chromosome segregation between daughter cells. To do this, we performed time-lapse microscopy on cells expressing mChy-PopZ and YFP-ParBI, acquiring images at 5-min intervals in order to observe events at high temporal resolution (Fig. 5A; Movie S8). Cells that inherited a bright focus of mChy-PopZ at the pole that had been the site of bud growth (indicated by the closed red stars in Fig. 5) were usually delayed in ParBI segregation relative to their siblings (indicated by open red stars) by an average time of 17 min (Fig. 5B, left box). Despite the delay in chromosome I segregation, cells that inherited mChy-PopZ relocated mChy-PopZ foci to the new growth pole by an average of 17 min before their siblings accumulated *de novo* mChy-PopZ at the growing pole (Fig. 5B, right box).

Directly comparing the accumulation of mChy-PopZ at the new pole to the initiation of chromosome I segregation shows that the former precedes the latter by an average of 19 min in cells that inherit mChy-PopZ (Fig. 5C, left box). The behavior of these cells indicates a departure from the C. crescentus model, in which polar accumulation of polar PopZ is triggered by the approach of the centromere (16). In contrast, A. tumefaciens daughter cells that did not inherit mChy-PopZ produced a distinct *de novo* focus of polar mChy-PopZ by an average of 15 min after the initiation of chromosome centromere segregation (Fig. 5C, right box). As segregating YFP-ParB-labeled centromeres took more than 20 min to move to the new cell pole (Fig. 5D), this cell type accumulates mChy-PopZ at the new pole during chromosome segregation, a timing that is similar to that of C. crescentus (17). Overall, our results show that the asymmetric distribution of polar regulatory factors that we (Fig. 3) and others (6, 7) have shown in A. tumefaciens is concomitant with the generation of daughter cells with different cell cycle timing.

C. crescentus also produces distinct daughter cell types with differential cell cycle timing, and in this species, the daughter cell that is delayed in chromosome replication is 21% shorter than its sibling (30). We observed a similar relationship between cell length and the timing of chromosome replication in A. tumefaciens (Fig. 5E). Cells that inherited polar foci of mChy-PopZ were a median of 12% shorter than their siblings at the time of cell division. Notably, the disparity in cell length was reduced to 5% when measured at the time of YFP-ParBI segregation, because the cells that inherited mChy-PopZ had more time to elongate due to the delay in chromosome duplication. For both cell types, we observed that longer cell length at the time of cell division correlated with shorter times to chromosome segregation (Fig. 5F). This implies that longer cells tend to have shorter cell cycles and will divide earlier than shorter cells, which will tend to divide later. Consequently, cells with lengths that deviate from the average tend to produce progeny that are closer to average. This is consistent with the idea that bacterial cells modulate their cell cycle time with respect to cell length in order to achieve cell length homeostasis (30).

## DISCUSSION

A. tumefaciens and C. crescentus PopZ have the same domain structure, with highly conserved N- and C-terminal regions flanking a middle section that is variable in length and amino acid sequence (23). In this work, we have shown that A. tumefaciens and C. crescentus both employ PopZ in chromosome segregation, indicating that some aspects of protein function are also conserved. In these species, the Δ*popZ* knockout results in untethered centromeres, and cell division often produces daughter cells that lack DNA.

During the A. tumefaciens cell cycle, the budding or so-called growth pole inherits a polar focus of mChy-PopZ. This is the same spatial and temporal position as the new pole in the C. crescentus cell cycle. In both species, the daughter cells that inherit these poles have similar characteristics in the next round of cell division. This cell type is shorter in length and delayed in chromosome segregation compared to its sibling, and it undergoes a polar transition event in which the new or growth pole changes to a terminally differentiated state prior to the next round of cell division. The siblings that inherit the nongrowing or old pole also have common characteristics, such as rapid reentry into the next round of cell division and the inheritance of a terminally differentiated pole. However, it is important to note that there are also significant differences between the A. tumefaciens and C. crescentus cell cycles. The distinct qualities of A. tumefaciens identified in this work include the nature of the polar transition (PopZ relocation), the late arrival of the chromosome I centromere relative to the accumulation of PopZ at the destination pole, and PopZ-independent anchoring of centromeres to old poles.

Another difference is that C. crescentus Δ*popZ* cells often divide very close to the new pole, creating minicells (14), whereas A. tumefaciens Δ*popZ* cells usually divide closer to midcell, producing daughters that are somewhat closer to normal size (23). The discrepancy is probably related to the machinery that determines the localization of the division plane. In C. crescentus, the placement of the Z-ring is inhibited by a centromere-associated protein called MipZ (31). A. tumefaciens lacks an MipZ homolog, but its genome encodes the components of the Min system, which blocks the formation of Z-rings as it oscillates between poles (32). It may be that the A. tumefaciens Min system operates in a manner that is not directly dependent on the localization of centromeres or PopZ.

While our observations of chromosome dynamics in A. tumefaciens thus far are limited to the centromere of chromosome I, it is important to note that our wild-type strain of A. tumefaciens contains three separate genetic elements. Chromosome I is a large circular chromosome with a conventional *par* segregation system, and there is also a linear chromosome of nearly equal size, as well as a smaller megaplasmid of <550 kb (33). The Ti virulence plasmid, which is not always present in natural populations (34), is not carried in the strains used for this study. All three of the replicons that are present include the genes for a RepABC-type replication and segregation mechanism, which is likely to have the same general properties of the *par* system (35). Their centromeric elements are known to be in close proximity to the cell poles (29), suggesting that their segregation also depends on PopZ, either directly or indirectly. A remaining question is whether the localization and dynamics of these other replicons are affected in the Δ*popZ* mutant. Our DAPI staining experiments suggest that this is the case, as many Δ*popZ* cells inherit very little total DNA. In comparison, DNA is distributed throughout the cytoplasm at all stages of cell division in wild-type cells. Notably, in time-lapse experiments of DAPI-stained Δ*popZ* cells, it appears that DNA is sometimes pulled out of a budding daughter cell in the direction opposite from normal chromosome segregation. This could be a result of the activity of the conserved FtsK/SpoIIIE DNA translocase, which helps to complete cell division by pumping DNA across the division plane in the direction of the chromosomal centromere (36). When unanchored or missegregated chromosomal centromeres become trapped on the wrong side of the division plane, FtsK/SpoIIIE can translocate the whole chromosome in the direction opposite its normal direction of travel (37).

We found that the asymmetric cell division in A. tumefaciens results in differential timing of chromosome replication between the two daughter cells. Although A. tumefaciens appears to be similar to C. crescentus in this regard, a surprising aspect of cell division in A. tumefaciens is that the relative timing of the arrival of PopZ at the new pole and the segregation of chromosome I is not the same in both daughter cells. Observations in C. crescentus suggest that the onset of polar localization of PopZ is triggered by ParA, a component of the *par* chromosome segregation machinery (16). In this species, ParA becomes increasingly concentrated at the new cell pole as it draws the centromere closer to its destination, and through direct interaction with PopZ, the increased concentration of ParA serves as a nucleation site for a polar focus of PopZ. This is consistent with what we observe in A. tumefaciens cells that inherit an old pole. However, in cells that inherit PopZ, we find that it relocates across the cell to the new pole several minutes before the onset of chromosome I segregation. The simplest explanation is that other replicons are segregated before chromosome I in this cell type, and this is sufficient to nucleate PopZ localization at the new pole. It is also possible that A. tumefaciens PopZ can be localized by another mechanism.

**FIG 6:**
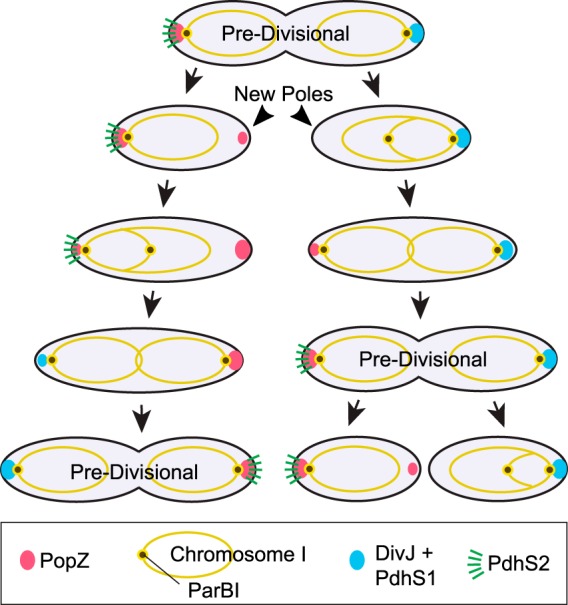
Model of chromosome I segregation and dynamic polar regulatory protein localization in A. tumefaciens. This model shows the relative positions of chromosome I, PopZ, and other polar regulatory proteins over the course of a normal A. tumefaciens cell cycle. Note that the daughter cells differ in polar inheritance and cell cycle progression, yet they produce a similar predivisional cell.

A. tumefaciens differs significantly from C. crescentus in the pattern of PopZ localization. In A. tumefaciens, only one of the daughter cells inherits a polar focus of PopZ, and instead of remaining in place, it undergoes a dramatic redistribution to the opposite cell pole during polar maturation (summarized in Fig. 6). Our analyses of histidine kinase localization patterns revealed that PopZ redistribution may be linked to the dynamic localization of polar regulators. The appearance of PdhS2 at the old pole preceded the loss of PopZ from this location, and PdhS2 polar foci disappeared after PopZ redistribution was complete. However, PdhS2 is quite unlikely to be playing a direct role in PopZ localization, since Δ*pdhS2* knockout strains exhibit normal growth and morphology (6). Instead, PdhS2 may respond to an upstream localization cue that also controls factors that regulate the distribution of PopZ. One such factor could be the polar transmembrane protein PodJ, which colocalizes with PopZ at the new pole (7) and is required for PopZ relocalization after cell division (19).

Overall, this study lends strong support to the idea that polar organization and cell cycle regulation in A. tumefaciens is significantly different from the C. crescentus model, even though the two related species use many of the same regulatory components. This is consistent with the finding that the essential cell cycle regulatory genes in A. tumefaciens and C. crescentus are overlapping but not identical (38), and that a key transcription factor in cell cycle-dependent gene expression controls different sets of genes in different Alphaproteobacteria species (39). We propose that the changes in polar regulatory networks that occurred during the evolution of Alphaproteobacteria followed the same basic set of principles that governed the rewiring of transcriptional networks in the evolution of ascomycete yeasts (40). In the yeast example, there is strong selective pressure to maintain working connections between sensory inputs and network outputs, but the complexity of the intervening mechanism allows changes in network connectivity that can become large over evolutionary time. The structure and logic of the network is therefore dictated by evolutionary history rather than an optimal design. In the future, it will be interesting to understand how changes in the organization of polar networks are related to the abundance of phenotypic diversity in Alphaproteobacteria.

## MATERIALS AND METHODS

### Cell culture.

Agrobacterium tumefaciens strain C58C1 (41), which is also known as Agrobacterium fabrum C58C1, and its derivatives were grown with aeration at 30°C in ATGN medium (42). The addition of iron salts was not necessary for robust growth. When appropriate, media were supplemented with antibiotics at the following concentrations (in micrograms per milliliter in liquid and solid medium, respectively): kanamycin (70 and 150), gentamicin (90 and 300), and spectinomycin (100 and 250). After initial strain construction, the Δ*popZ* lesion was maintained without antibiotic selection. To induce expression from pSRK plasmids, isopropyl-β-d-thiogalactopyranoside (IPTG) was added at 300 μM final concentration for 2 to 3 h prior to analysis. When DAPI was used to stain DNA, it was added to a final concentration of 2 μg/ml for 5 min prior to analysis.

### Strain construction.

Lists of strains and plasmids used in this work are provided in Tables S1 and S2 in the supplemental material. To create the Δ*popZ* strain (GB1163), we used a standard allelic replacement technique (43) to replace the *popZ* coding sequence in A. tumefaciens strain C58C1 with a cassette that confers resistance to spectinomycin. We complemented the Δ*popZ* lesion in GB1163 by introducing plasmid pGB1178, which integrated at the *popZ* promoter and resulted in the expression of mChy-PopZ from the native *popZ* locus (strain GB1158). To express a photoconvertible fluorescent version of PopZ, we placed the *meos3.2-popZ* coding sequence into a broad-host-range plasmid (pSRK) that enables expression from an IPTG-inducible promoter (25). To express fluorescently tagged histidine kinase proteins, we placed monomeric superfolder GFP-tagged coding sequences downstream of the C. crescentus pvanA promoter in a broad-host-range plasmid (44). In A. tumefaciens, protein expression from this plasmid is not inducible by vanillate, as it is in C. crescentus, but is sufficient to drive low levels of protein expression without any inducer (our observations). To visualize ParBI localization, we placed the *eyfp-parBI* coding sequence into pSRK, which enabled IPTG-inducible expression. Details on plasmid construction are available in the supplemental material.

### Wide-field fluorescence microscopy and image analysis.

For microscopy and image analysis, cells from log phase cultures were immobilized on a 1% agarose pad containing ATGN medium. Live-cell imaging was performed at room temperature using a motorized Zeiss Axio Imager Z2 epifluorescence microscope equipped with a Hamamatsu Orca-Flash4.0 sCMOS camera and a Plan-Apochromat 100×/1.46-numeric-aperture oil Ph3 objective. Zeiss filter sets 49DAPI, 38HE, 46HE, and 63HE were used to acquire fluorescent images of DAPI, msfGFP/mEOS3.2, enhanced YFP, and mCherry, respectively. Cell lengths and centromere positions were calculated by drawing segmented, spline-fit lines over composite images at 300% zoom in ImageJ and tabulating them with the ROI Manager tool. Cell types were quantified using the ImageJ cell counter plug-in.

## Supplementary Material

Supplemental material
