# Supplementary material for: Polar Organizing Protein PopZ Is Required for Chromosome Segregation in Agrobacterium tumefaciens
Source: J Bacteriol. 2017 Aug 8;199(17):e00111-17. doi: 10.1128/JB.00111-17 (PMC5553026; doi:10.1128/JB.00111-17)
Supplement: Supplemental material [file supp_199_17_e00111-17__index.html]

Supplemental material 

# Polar Organizing Protein PopZ Is Required for Chromosome Segregation in Agrobacterium tumefaciens

## Supplemental material

- Supplemental file 1 -

  Supplemental text, movie legends, and Tables S1 (Strains used) and S2 (Plasmids used)

  PDF, 200K
- Supplemental file 2 -

  Movie S1 (Time-lapse movie of growing Δ*popZ* cells)

  AVI, 10M
- Supplemental file 3 -

  Movie S2 (Time-lapse movie of cells expressing mChy-PopZ)

  AVI, 1.2M
- Supplemental file 4 -

  Movie S3 (Time-lapse movie of a dividing cell expressing PdhS1-GFP and mChy-PopZ)

  AVI, 728K
- Supplemental file 5 -

  Movie S4 (Time-lapse movie of dividing cell expressing PdhS2-GFP and mChy-PopZ)

  AVI, 1.9M
- Supplemental file 6 -

  Movie S5 (Time-lapse movie of growing Δ*popZ* cells expressing PdhS1-GFP)

  AVI, 5.6M
- Supplemental file 7 -

  Movie S6 (Time-lapse movie of growing Δ*popZ* cells expressing PdhS2-GFP)

  AVI, 800K
- Supplemental file 8 -

  Movie S7 (Time-lapse movie of growing Δ*popZ* cells expressing YFP-ParBI)

  AVI, 14M
- Supplemental file 9 -

  Movie S8 (Time-lapse movie of dividing cells expressing YFP-ParBI)

  AVI, 4.9M
