# Supplementary material for: Polar Organizing Protein PopZ Is Required for Chromosome Segregation in Agrobacterium tumefaciens
Source: J Bacteriol. 2017 Aug 8;199(17):e00111-17. doi: 10.1128/JB.00111-17 (PMC5553026; doi:10.1128/JB.00111-17)
Supplement: Supplemental material [file JB.00111-17_zjb999094510s1.pdf]

SUPPLEMENTARY MATERIAL:

The polar organizing protein PopZ is required for chromosome segregation in *Agrobacterium tumefaciens*

Authors: Haley Ehrle*, Jacob Guidry*, Rebecca Iacovetto*, Anne Salisbury*, DJ Sandidge*, and Grant Bowman^
Department of Molecular Biology, University of Wyoming, Laramie WY 82071
*these authors contributed equally to this work.
^to whom correspondence should be addressed.

SUPPLEMENTARY METHODS:

Plasmid Construction:
E. coli strain DH5α was used for molecular cloning. For strain construction, plasmids were introduced into *Agrobacterium* by mating or electroporation, using the techniques described by Morton and Fuqua (1).

To create plasmid pGB1161, pNPTS1318 (2) was linearized with enzymes SpeI and NheI. One-step isothermal cloning (3) was used to insert three fragments: the Ω cassette, which confers spectinomycin/streptomycin resistance, was originally from pHP45-omega (4), and was modified to have BamHI flanking sites, and this was cloned in between two 500 bp sections from either flank of the *Agrobacterium* popZ coding sequence, which were amplified from strain C58C1 using the following primers:
5'Leflk_AtPo_ITC_SpeI: attacgccaagctacgtaatacgactcactagTgccaaggcaactgtctat
3'Leflk_AtPo_ITC_EcoRV: CTTGCTCAATCAATCACCGGATCCagatatcaatccccggtttctact
5'Rtflk_AtPo_ITC_EcoRI: AGCTTGCTCAATCAATCACCGGATCCacgaattcTccgcaggctacaatcgct
3'Rtflk_AtPo_ITC_NheI: cggagacgcgtcacggccgaaGCTAGCtggttttccatcagcttgc

To create plasmid pGB1178, plasmid pMCS4 (5) was linearized with EcoRI and NheI. One-step isothermal cloning (3) was used to insert two fragments: the *popZ* promoter was amplified from strain C58C1 with the following primers:
pMCS4_5'AtPoLeflk_ITC_EcoRI_FOR: ccttaagatctcgagctccggagaattcTgccaaggcaactgtctat
3'leftflank_5'mcherry_REV_ITC: TCGCCCTTGCTCATatcaatccccggtttctac
And the *mchy-popZ* coding sequence, which was amplified from plasmid pGB1157 using the following primers:
3'leftflank_5'mCherry_FOR_ITC: aaccggggattgatATGAGCAAGGGCGAGGAGG
3'mCheAtPo_pMCS4_REV_ITC: gtggatcccccgggctgcagctagcTTAgcggcgcgagccg
To create plasmid pGB1157, plasmid pRVMCS2 (5) was linearized with enzymes NdeI and BglII. One-step isothermal cloning was used to insert two fragments: *mchy* was amplified using the following primers:
pRV_ITC_5'mCherry: ccgaaccacgatgcgaggaaacgcatatgGTGAGCAAGGGCGAGGAG
3'mCherry_ITC_5'AtPopZ: TACAAGtccggcggtggtggcATGgctcagccaagtgtcgc

And popZ was amplified from strain C58C1 with the following primers:
5'AtPopZ_ITC_3'mCherry: gcCATgccaccaccgccggaCTTGTACAGCTCGTCCATGCCG
pRV_BglIIITC_3'AtPopZ: tgcagctagcaccggtacgtagatctTTAgcggcgcgagccgc

To create plasmid pGB1249, plasmid pSRKgm (6) was linearized with enzymes NdeI and HindIII. One-step isothermal cloning (3) was used to insert two fragments: mEos3.2 was amplified from Addgene plasmid # 54550 (a gift from Michael Davidson) using the following primers:
pSRK_mEOS_FOR: GCGGATAACAATTTCACACAGGAAACAGCATatgAGTGCGATTAAGCCAGA
5'AtPopZ_ITC_3'mEOS: gctgagcCATggatccagatccTCGTCTGGCATTGTCAGG
And popZ was amplified from a PCR fragment containing the popZ coding sequence using the following primers:
3'mEOS_ITC_5'AtPopZ: CCAGACGAggatctggatccATGgctcagccaagtgtcgc
ATpopZ_pSRK_REV: CCCTCGAGGTCGACGGTATCGATAAGCTTttagcggcgcgagccgc

To create plasmid pGB1200, plasmid pRVMCS2 (5) was linearized with enzymes NdeI and NheI. One-step isothermal cloning (3) was used to insert two fragments: the *pdhS1* coding sequence was amplified from strain C58C1 with the following primers:
5'prVMCS2_PdhS1_base+tailA: ccgaaccacgatgcgaggaaacgcatATGCCCGCCGTCCAATATC
3'PdhS1_GFPbase+tailA: CACCTTTACTgctagtggtaccGTTGGCAAGAACCCGCTG
And monomeric superfolder GFP (msfgfp) coding sequence (Pedelacq et al 2007 REF) was amplified using the following primers:
5'PdhS1_GFP_base+tailA: TCTTGCCAACggtaccactagcAGTAAAGGTGAAGAACTGTTCACC
3'GFP_pRVMCS2_base+tailA: gtggatcccccgggctgcagctagcTCATTTGTAGAGTTCATCCATGCCG

To create plasmid pGB1199, plasmid pRVMCS2 (5) was linearized with enzymes NdeI and NheI. One-step isothermal cloning (3) was used to insert two fragments: the *divJ* coding sequence was amplified from strain C58C1 with the following primers:
5'pRVMCS2_DivJ_base+tail: cgaaccacgatgcgaggaaacgcatATGAGAGAAAAAGCGGTC
3'Divj_GFPbase+tailc: CTTCACCTTTACTgctagtggtaccGGCGATTTTCGCTTTCG
And monomeric superfolder GFP (*msfgfp*) coding sequence (Pedelacq et al 2007 REF) was amplified using the following primers:
5'DivJ_GFP_base+tailc: CGAAAATCGCCggtaccactagcAGTAAAGGTGAAGAACTGTT
3'GFP_pRVMCS2_base+tail+trim: ggatcccccgggctgcagctagcTCATTTGTAGAGTTCATCCATGC

To create plasmid pGB1171, plasmid pRVMCS2 (5) was linearized with enzymes NdeI and NheI. One-step isothermal cloning (3) was used to insert two fragments: the *pdhS2* coding sequence was amplified from strain C58C1 with the following primers:
5'pRVMCS2_atPdhS2: ccacgatgcgaggaaacgCATatgAGTAAAAGCGTCAGCACG
3'atPdhS2_msfGFP: CCTTTACTgctagtggtaccGGCGAAAGACCGCCGGCG
And monomeric superfolder GFP (*msfgfp*) coding sequence (Pedelacq et al 2007 REF) was amplified using the following primers:

5'atPdhS2_msfGFP: TTCGCCggtaccactagcAGTAAAGGTGAAGAACTGTTC
3'msfGFP_pRVMCS2: gtggatcccccgggctgcagctagcTCATTTGTAGAGTTCATCCATGC

To create plasmid pGB1207, plasmid pSRKkm (6) was linearized with the enzymes NdeI and HindIII. One-step isothermal cloning (3) was used to insert the *yfp-parBI* coding sequence, which was amplified from plasmid pGB1190 with the following primers:
pSRK_eYFP_FOR: GCGGATAACAATTTCACACAGGAAACAGCATatgAGCAAGGGCGAGGAG
ParB2828_pSRK_REV: CCCTCGAGGTCGACGGTATCGATAAGCTTttaTTTCTGCTCCAGCAGCC
To create plasmid pGB1190, pNPTS1318 (Ried and Collmer REF) was linearized with the enzymes EcoRV and NheI. One-step isothermal cloning (Gibson REF) was used to insert three fragments: The left flank of the parBI coding sequence was amplified from strain C58C1 with the following primers:
pNPTS138_2828ParBLftFlnk_FOR:
gccaagcttctctgcaggatatctggCCGAATTATCGCAGTTGCGAAC
2828ParBLftFlnk_eYFP_REV_II: CTCGCCCTTGCTCATaagatattactccatacagtccgc
And the *eyfp* coding sequence was amplified with the following primers:
2828ParBLftFlnk_eYFP_FOR_II:gactgtatggagtaatatcttATGAGCAAGGGCGAGGAG
eYFP_2828ParB_REV:cgaaagatcatcactCATggatccagatccCTTGTACAGCTCGTCCATGC
And the *parBI* coding sequence was amplified from strain C58C1 with the following primers:
eYFP_2828ParB_FOR:GCTGTACAAGggatctggatccATGagtgatgatctttcgaagcgtc
2828ParB_pNPTS138_NheI_REV:cggagacgcgtcacggccgaagTTATTTCTGCTCCAGCAGCCG

SUPPLEMENTARY REFERNCES

1. **Morton ER**, **Fuqua C**. 2012. Genetic manipulation of *Agrobacterium*. Curr Protoc Microbiol Chapter 3: Unit 3D.2.
2. **Ried JL**, **Collmer A**. 1987. An nptI-sacB-sacR cartridge for constructing directed, unmarked mutations in gram-negative bacteria by marker exchange-eviction mutagenesis. Gene **57**:239– 246.
3. **Gibson DG**, **Young L**, **Chuang R-Y**, **Venter JC**, **Hutchison CA**, **Smith HO**. 2009. Enzymatic assembly of DNA molecules up to several hundred kilobases. Nat Methods **6**:343–345.
4. **Prentki P**, **Krisch HM**. 1984. *In vitro* insertional mutagenesis with a selectable DNA fragment. Gene **29**:303–313.
5. **Thanbichler M**, **Iniesta AA**, **Shapiro L**. 2007. A comprehensive set of plasmids for vanillate- and xylose-inducible gene expression in *Caulobacter crescentus*. Nucleic Acids Res **35**:e137.
6. **Khan SR**, **Gaines J**, **Roop RM**, **Farrand SK**. 2008. Broad-host-range expression vectors with tightly regulated promoters and their use to examine the influence of TraR and TraM expression on Ti plasmid quorum sensing. Appl Environ Microbiol **74**:5053–5062.

FIGURE LEGENDS FOR SUPPLEMENTARY MOVIES M1-M8:

Supplementary Movie M1:
Strain: *popZ::Δ* (GB1163)
Time: 14 frames, 20 minutes per interval.
Description: Time-lapse movie of growing Δ*popZ* cells. The phase contrast image is shown in grayscale.

Supplementary Movie M2:
Strain: *popZ::mchy-popZ* (GB1185)
Time: 17 frames, 15 minutes per interval.
Description: Time-lapse movie of cells expressing mChy-PopZ (red) from the native promoter. The fluorescence image is overlayed on the phase contrast image in grayscale. In the two cells on the left, PopZ accumulates at the new cell poles after cell division. In the cell at right, a bright focus of mChy-PopZ appears to accumulate in the area of the divison plane before the cells have clearly divided.

Supplementary Movie M3:
Left Panel:
Strain: *popZ::mchy-popZ ;* pRVMCS2 *+ pdhS1-msfgfp* (GB1205)
Time: 11 frames, 20 minutes per interval.
Description: Time-lapse movie of a dividing cell expressing PdhS1-GFP (green) and mChy-PopZ (red). The two fluorescence images are overlayed on the phase contrast image in grayscale. PdhS1-GFP localizes stably to the old cell pole after mChy-PopZ has relocated to the new pole.
Right Panel:
Strain: *popZ::mchy-popZ ;* pRVMCS2 *+ divJ-msfgfp* (GB1196)
Time: 11 frames, 20 minutes per interval.
Description: Time-lapse movie of a dividing cell expressing DivJ-GFP (green) and mChy-PopZ (red). The two fluorescence images are overlayed on the phase contrast image in grayscale. DivJ-GFP localizes stably to the old cell pole after mChy-PopZ has relocated to the new pole.

Supplementary Movie M4:
Strain: *popZ::mchy-popZ ;* pRVMCS2 *+ pdhS2-msfgfp* (GB1193)
Time: 20 frames, 20 minutes per interval.
Description: Time-lapse movie of a dividing cell expressing PdhS2-GFP (green) and mChy-PopZ (red). The two fluorescence images are overlayed on the phase contrast image in grayscale. PdhS2-GFP appears at the transitional pole just before the onset of mChy-PopZ relocation. PdhS2-GFP disappears from the old pole after the focus of mChy-PopZ has gone.

For clarity, three different views of the movie are shown. In the left frames, only the PdhS2-GFP fluorescence signal is shown (in green), overlayed on the phase contrast image in grayscale. In the center frames, fluorescent signals from PdhS2 (green) and mChy-PopZ (red) are overlayed on the phase contrast image in grayscale. In the right frames, only the mChy-PopZ fluorescence signal is shown (in red), overlayed on the phase contrast image in grayscale.

Supplementary Movie M5:
Left Panel:
Strain: *popZ::Δ ;* pRVMCS2 *+ pdhS1-msfgfp* (GB1206)
Time: 14 frames, 20 minutes per interval.
Description: Time-lapse movie of growing Δ*popZ* cells expressing PdhS1-GFP (green). The fluorescence image is overlayed on the phase contrast image in grayscale. PdhS1-GFP remains stably associated with poles that do not appear to be growing.
Right Panel:
Strain: *popZ::Δ ;* pRVMCS2 *+ divJ-msfgfp* (GB1198)
Time: 14 frames, 20 minutes per interval.
Description: Time-lapse movie of growing Δ*popZ* cells expressing DivJ-GFP (green). The fluorescence image is overlayed on the phase contrast image in grayscale. DivJ-GFP remains stably associated with poles that do not appear to be growing.

Supplementary Movie M6:
Strain: *popZ::Δ ;* pRVMCS2 *+ pdhS2-msfgfp* (GB1170)
Time: 24 frames, 10 minutes per interval.
Description: Time-lapse movie of growing Δ*popZ* cells expressing PdhS2-GFP (green). The fluorescence image is overlayed on the phase contrast image in grayscale. PdhS2 is transiently localized to some poles, and sometimes disappears from a pole after cell division.

Supplementary Movie M7:
Strain: *popZ::Δ ;* pRVMCS2 *+* pSRKkm + *eyfp-parBI* (GB1210)
Left Panel:
Time: 18 frames, 20 minutes per interval.
Description: Time-lapse movie of growing Δ*popZ* cells expressing YFP-ParBI , which labels the chromosome I centromere (yellow). The fluorescence image is overlayed on the phase contrast image in grayscale. Chromosome I is improperly segregated in Δ*popZ* cells. Following centromere duplication, the centromere that is moved towards the new pole usually fails to reach its target. In some cases the division plane forms between the mis-segregated centromere and the new pole, creating a daughter cell with no chromosome I centromere. The culture also includes small, non-growing cells.
Right Panel:
Time: 12 frames, 20 minutes per interval.

Description: Time-lapse movie of growing Δ*popZ* cells expressing YFP-ParBI, which labels the chromosome I centromere (yellow). Total DNA is was labeled with DAPI (blue) prior to analysis. The fluorescence images are overlayed on the phase contrast image in grayscale. Following centromere duplication, the centromere that is moved towards the new pole often fails to reach its target. In these cases, the division plane forms between the mis-segregated centromere and the new pole, creating a daughter cell with no chromosome I centromere and very little total DNA. Notably, the DNA appears to get pumped out of the cell in the minutes before cell division. Those cells that have no chromosome I centromere and little total DNA do not grow.

Supplementary Movie M8:
Strain: *popZ::mchy-popZ ;* pSRKkm + *eyfp-parBI* (GB1209)
Time: 40 frames, 5 minutes per interval.
Description: Time-lapse movie of dividing cells expressing YFP-ParBI, which labels the chromosome I centromere (green), and mChy-PopZ (red). The fluorescence image is overlayed on the phase contrast image in grayscale. In both cases, the cell that inherits the bright focus of PopZ (Z+) is delayed in chromosome I segregation relative to its sibling (Z-). In Z+ cells, PopZ is relocated to the new pole well before centromere I segregation. In Z- cells, a bright focus of mChy-PopZ appears during or soon after centromere I segregation.

For clarity, three different views of the movie are shown. In the left frames, only the YFP-ParB fluorescence signal is shown (in green), overlayed on the phase contrast image in grayscale. In the center frames, fluorescent signals from YFP-ParB (green) and mChy-PopZ (red) are overlayed on the phase contrast image in grayscale. In the right frames, only the mChy-PopZ fluorescence signal is shown (in red), overlayed on the phase contrast image in grayscale.

Supplementary Table 1. *Agrobacterium* Strains used in this study.

***Agrobacterium fabrum* strains**	**Relevant genotype/description**	**Construction, source or reference**
Wildtype (C58C1)	derivative of C58, cured of pTiC58 virulence plasmid	Van Larebeke et al. (1974)
GB1163	*popZ::Δ* (in C58C1)	This study, as described in Materials and Methods
GB1185	*popZ::mchy-popZ* (in C58C1)	pGB1178 mated into GB1163 (this study)
GB1259	C58C1 ; pSRKgm + *meos3.2-popZ*	pGB1249 electroporated into C58C1 (this study)
GB1205	*popZ::mchy-popZ ;* pRVMCS2 *+ pdhS1-msfgfp*	pGB1200 electroporated into GB1185 (this study)
GB1196	*popZ::mchy-popZ ;* pRVMCS2 *+ divJ-msfgfp*	pGB1199 electroporated into GB1185 (this study)
GB1193	*popZ::mchy-popZ ;* pRVMCS2 *+ pdhS2-msfgfp*	pGB1171 electroporated into GB1185 (this study)
GB1206	*popZ::Δ ;* pRVMCS2 *+ pdhS1-msfgfp*	pGB1200 electroporated into GB1163 (this study)
GB1198	*popZ::Δ ;* pRVMCS2 *+ divJ-msfgfp*	pGB1199 electroporated into GB1163 (this study)
GB1170	*popZ::Δ ;* pRVMCS2 *+ pdhS2-msfgfp*	pGB1171 electroporated into GB1163 (this study)
GB1209	*popZ::mchy-popZ ;* pSRKkm + *eyfp-parBI*	pGB1207 electroporated into GB1185 (this study)
GB1210	*popZ::Δ ;* pSRKkm + *eyfp-parBI*	pGB1207 electroporated into GB1163 (this study)

1. **Van Larebeke N**, **Engler G**, **Holsters M**, **Van den Elsacker S**, **Zaenen I**, **Schilperoort RA**, **Schell J**. 1974. Large plasmid in Agrobacterium tumefaciens essential for crown gall-inducing ability. Nature **252**:169–170.

Supplementary Table 2. Plasmids used in this study.

Name	Description	Backbone	Source
pGB1161	For creating Δ*popZ* strain by double recombination	pNPTS138	This study
pGB1178	For integrating P_{popZ}-*mchy-popZ* at Δ*popZ* locus	pMCS4	This study
pGB1249	For inducible expression of *meos3.2-popZ*	pSRKgm	This study
pGB1200	For constitutive low-level expression of *pdhS1-msfgfp*	pRVMCS2	This study
pGB1199	For constitutive low-level expression of *divJ-msfgfp*	pRVMCS2	This study
pGB1171	For constitutive low-level expression of *pdhS2-msfgfp*	pRVMCS2	This study
pGB1207	For inducible expression of *eyfp-parBI*	pSRKkm	This study
pNPTS138	Host plasmid for double recombination		Ried and Collmer, 1987 (1)
pMCS4	Host plasmid for chromosomal integration		Thanbichler *et al.*, 2007 (2)
pSRKgm	Broad host-range plasmid for inducible protein expression		Khan *et al.*, 2008 (3)
pRVMCS2	Broad host-range plasmid with *Caulobacter vanA* promoter		Thanbichler *et al.*, 2007 (2)
pSRKkm	Broad host-range plasmid for inducible protein		Khan *et al.*, 2008 (3)

	expression		

1. **Ried JL**, **Collmer A**. 1987. An nptI-sacB-sacR cartridge for constructing directed, unmarked mutations in gram-negative bacteria by marker exchange-eviction mutagenesis. Gene **57**:239–246.
2. **Thanbichler M**, **Iniesta AA**, **Shapiro L**. 2007. A comprehensive set of plasmids for vanillate- and xylose-inducible gene expression in Caulobacter crescentus. Nucleic Acids Res **35**:e137.
3. **Khan SR**, **Gaines J**, **Roop RM**, **Farrand SK**. 2008. Broad-host-range expression vectors with tightly regulated promoters and their use to examine the influence of TraR and TraM expression on Ti plasmid quorum sensing. Appl Environ Microbiol **74**:5053–5062.
